# Predicting viral load suppression by self-reported adherence, pharmacy refill counts and real time medication monitoring among people living with HIV in Tanzania

**DOI:** 10.1186/s12981-022-00475-y

**Published:** 2022-11-15

**Authors:** Kennedy M. Ngowi, Linda Minja, I. Marion Sumari-de Boer, Rob E. Aarnoutse, Lyidia Masika, Mirjam A. G. Sprangers, Francis M. Pima, Blandina T. Mmbaga, Peter Reiss, Pythia T. Nieuwkerk

**Affiliations:** 1 Kilimanjaro Clinical Research Institute, Moshi, United Republic of Tanzania; 2 Department of Medical Psychology, Amsterdam University Medical Center, University of Amsterdam, Amsterdam, The Netherlands; 3 Amsterdam Institute for Global Health and Development, Amsterdam, the Netherlands; 4 Radboudumc, Radboud Institute for Health Sciences and Department of Pharmacy, Nijmegen, The Netherlands; 5 Kilimanjaro Christian Medical Centre, Moshi, United Republic of Tanzania; 6 Kilimanjaro Christian Medical University College, Moshi, United Republic of Tanzania; 7 Department of Global Health, and Amsterdam Institute for Global Health and Development Amsterdam UMC, University of Amsterdam, Amsterdam, The Netherlands

## Abstract

**Introduction:**

Monitoring of adherence to antiretroviral treatment (ART) is of utmost importance to prevent treatment failure. Several measures to monitor adherence have been applied in low-resource settings and they all have pros and cons. Our objective was to examine whether any of the following adherence measures is a better predictor of participants’ viral load suppression: (1) self-report, (2) pharmacy refill count, (3) Real Time Medication Monitoring (RTMM), (4) a combination of self-report and pharmacy refill count or (5) all three adherence assessment methods combined.

**Methodology:**

This was a post-hoc analysis of data from our 48-week REMIND-HIV randomized controlled trial in which adherence to ART was measured using self-report, pharmacy refill counts and RTMM among ART-experienced adults living with HIV subjectively judged to be nonadherent to ART. For each adherence measure, we calculated sensitivity, specificity, positive predictive value (PPV) and negative predictive value (NPV) for predicting virological failure defined as a viral load (VL) of > 20 copies/mL. To determine at which percentage of adherence the prediction was strongest, we evaluated adherence cut-offs of 80%, 85%, 90%, 95% and 100% using receiver operating characteristic (ROC) curves. VL data were obtained after 48 weeks of follow-up in the trial.

**Results:**

A total of 233 people living with HIV (PLHIV) were included in this analysis. When comparing the ability of self-reported adherence with pharmacy refill count and RTMM adherence to predict viral load > 20 copies/ml, self-reported adherence had the lowest sensitivity, ranging from 6 to 17%, but the highest specificity, ranging from 100 to 86%, depending on cut-off values from 80 to 100%. Area under the ROC curves (AUC) were 0.54 for RTMM, 0.56 for pharmacy refill count and 0.52 for self-report, indicating low discriminatory capacity for each of the adherence measures. When we combined the self-report and pharmacy refill count measures, sensitivity increased, ranging from 28 to 57% but specificity decreased, ranging from 83 to 53%. When all three measures were combined, we observed the highest value of sensitivity, ranging from 46 to 92%, and PPV, ranging from 32 to 36%, at high cut-offs ranging from 80 to 100%. Upon combination of three adherence measures, the AUC increased to 0.59.

**Conclusion:**

Our results show that adherence assessed exclusively by self-report, pharmacy refill count or RTMM were insufficiently sensitive to predict virologic failure. Sensitivity markedly improved by combining all three measures, but the practical feasibility of such an approach would need to be studied.

## Introduction

In 2019, an estimated 24 million people were living with HIV (PLHIV) in Sub-Saharan Africa, of whom approximately 73% were adults 15–49 years of age, approximately 75% of whom were on antiretroviral treatment (ART) [1]. Maintaining high rates of adherence to ART is vital to maintain viral suppression and reduce morbidity, disease progression and mortality among PLHIV in a sustained manner [2–5]. The level of adherence to ART required to prevent virological failure and the emergence of antiretroviral drug resistance was previously considered to be at least 95% [6]. However, more recent studies suggest that current regimens are more forgiving of missing doses, with levels of 80% adherence potentially being sufficient [3]. Maintaining consistently high adherence levels over long time periods has been challenging for PLHIV. This is due, among others, to medication fatigue and dissatisfaction with HIV consultations provided at the clinic [7, 8]. Commonly used adherence assessment methods applied by clinicians in low-resource settings are self-reported adherence, pill counts and pharmacy refill counts [9, 10]. These methods are often used in standard clinical practice to support meaningful discussion about adherence between PLHIV and health care providers [10]. However, due to recall and social desirability bias, self-report methods tend to overestimate PLHIV’s actual adherence levels, whereas pill counts and pharmacy refill counts can easily be manipulated and may be too cumbersome to perform in routine clinical practice [11].

Several alternative adherence monitoring tools have been recommended to overcome these drawbacks including digital adherence tools (DAT) [12], which make use of mobile phone communication. With the widespread use of mobile technology in Sub-Sahara African countries, adherence strategies deployed by mobile phones have the chance to reach a large user audience [13]. An example of such a strategy is the Wisepill^®^ device for real-time medication monitoring (RTMM). RTMM records the date and time of each opening of a medication box and is thus less susceptible to overestimating medication adherence than self-report and pill counts [14]. However, RTMM has its own technical challenges due to the fact that it relies on a battery in the device and on network availability in order to send a signal about an opening of the box to a server as a reflection of actual medication intake [2, 15]. As a result, inconsistent capturing of actual doses missed was reported in several studies investigating the use of RTMM [16–19]. Moreover, RTMM may underestimate actual adherence levels if participants do not ingest the pills directly from the device, but for example put retrieved pills in their pockets, so-called pocket dosing [20].

Previous studies have investigated which of the adherence assessment methods self-report, pharmacy refill count, or RTMM, may be the best predictor of virological suppression [21–24]. However, to our knowledge, there is limited evidence of the ability of those adherence methods to predict viral suppression when combined in a low income setting. Therefore, as part of our REMIND-HIV trial [25], the objective of this study was to examine whether any of the following adherence measures is a better predictor of PLHIVs’ viral suppression: (1) self-report, (2) pharmacy refill counts, (3) RTMM, (4) a combination of self-report and pharmacy refill count or (5) all three adherence assessment methods combined.

## Methods

We conducted a post-hoc analysis of part of the data from the randomized controlled REMIND-HIV trial, in which PLHIV had been randomly allocated to (1) RTMM, (2) Short Message Service (SMS) reminder texts or (3) standard of care and followed for 48 weeks. Details of the trial have been described elsewhere [25]. The study was approved by the College Research and Ethical Review Committee (CRERC) of Kilimanjaro Christian Medical University College (KCMUCo) and the National Health Research Ethics Sub-Committee (NatHREC) of the National Medical Research Institute (NIMR) of Tanzania. The trial was registered at the Pan African Clinical Trials Registry under PACTR201712002844286.

### Study population

Participants were recruited from two sites, which were Kilimanjaro Christian Medical Centre (KCMC) and Majengo Health Centre, both located in Moshi, Tanzania. PLHIV were approached by study nurses during a common clinic visit. Informed consent was obtained from all participants followed by screening for eligibility. The inclusion requirements were: (1) 18–65 years of age, (2) receiving antiretroviral treatment for at least 6 months, (3) subjectively judged by a nurse counsellor to be poorly adherent to medication, based on missed clinic visits, returning excess leftover medication, and/or having continuously high viral loads, (4) able to read and write and (5) able and willing to provide consent to study participation. We excluded participants if they (1) were admitted to the hospital and/or (2) participated in similar studies investigating digital adherence tools.

### Study Procedures

After obtaining informed consent from participants, study nurses interviewed participants and completed a screening form, containing inclusion and exclusion criteria, demographics, medical history, HIV history and times of usual ART intake. A secured web-based electronic data capture software system (REDcap) was used to collect and manage data. RedCap supports data validation, has an auditing trail and allows for data verification [26]. After completion of screening, the data manager performed randomization in REDcap using block randomization, stratified by gender and study site. Participants were randomized in one of three arms, RTMM, SMS or control arm, at a 1:1:1 ratio [25]. Participants were expected to attend the clinic every 2 months, according to standard care [27]. At each clinic visit, adherence was recorded through self-report, pharmacy refill count and, in the RTMM arm, additionally through RTMM. Participants were followed for 48 weeks. Viral load was measured at baseline and at the last week 48 study visit. For the present study, adherence and viral load data obtained at the week 48 study visit are used. Adherence measures considered the period since the last study visit preceding week 48. We did not include adherence data from the full study follow-up due to incompleteness of the data during earlier visits, though we considered leftover medication from the before-last visit.

### Adherence measures.

#### Self-reported adherence

Self-reported (SR) adherence was measured using a questionnaire that was administered during a face-to-face interview by study nurses at each study visit. The questionnaire included two adherence questions: (1) ‘How many pills do you take per day?’ and (2) ‘How many pills did you miss in the past month?’ We calculated adherence taking the number of swallowed pills divided by the number of prescribed pills using the following formula as described previously [28]. We calculated adherence as follows:

$${\text{Self - reported adherence in the past month}} = \left\{ {\frac{{\left[ {\left( {{\text{Number of days in the past month }} \times {\text{ pills to take per day}}} \right) - \left( {\text{missed pills}} \right)} \right]}}{{\left( {{\text{number of days in the past month }} \times {\text{ pills to take per day}}} \right)}}} \right\} \, \times { 1}00\%$$

#### Pharmacy refill counts (PR)

A case report form was administered face to face to record pharmacy refill data at each study visit. The study pharmacist recorded the number of pills dispensed during the previous visit by asking ‘How many pills were given to you at the previous visit?’ while checking the medical file for the same information. In addition, the left-over pills returned during the previous and current visit were counted. For the participants who did not return pills, we asked to recall the number of pills that were left at home. In case leftover pills were unknown, our assumption was that all pills had been taken as prescribed in during the previous visit. Adherence was calculated as follows:

$${\text{Pharmacy - refill adherence}}: \, \frac{{\left( {\left( {{\text{pills dispensed at previous visit }} + {\text{ returned pills at previous visit}}} \right) \, - {\text{ returned pills at current visit}}} \right)}}{{\left( {{\text{number of days between visits}}*{\text{number of pills to take per day}}} \right)}}\;*\;{1}00\% .$$

Assuming that levels higher than 100% were representing 100%, we truncated maximum adherence at 100%.

#### RTMM adherence

Participants in the RTMM arm were given a Wisepill^®^ RTMM device to monitor their medication intake in real time. When the device is opened, information including the time stamp is wirelessly sent using General Packet Radio Services (GPRS) to a secured web-based central database. Each opening was recorded, which was taken as a sign that the participant ingested the dose. If the box was not opened on time (agreed time between participant and healthcare provider), the participant received a short message service (SMS) text on his/her mobile phone which acted as a reminder to take medication.

Adherence levels were calculated at the 48-week follow-up visit of the study.

$${\text{Adherence}} = \, \left( {\frac{{\text{Number of openings over a given period}}}{{{\text{number of expected openings }}\left( {\text{based on prescription of number of dosing moments per day}} \right)}}} \right)\,*\,{1}00\%$$

#### Virological failure

HIV viral load data was obtained at 48 weeks of follow-up. The Tanzanian HIV guidelines direct health care workers to act once someone has 1000 copies/mL i.e. to provide enhanced adherence counselling or switch treatment [27, 29]. However, laboratory equipment in our study sites can determine viral load as low as 20 copies/ml. Therefore, plasma HIV RNA < 20 copies/ml was defined as virologically suppressed, while plasma HIV RNA < 1000 copies/ml was categorized as stable and plasma HIV RNA > 1000 copies/mL was categorized as unstable. As the trend in analyses of both cut-off values were the same, to answer our objective, we only described a viral load level > 20copies/mL as representative of virological failure.

#### Statistical analysis

Statistical analyses were conducted with Stata v.15. In the analyses, we included all participants who had a viral load measurement at week 48. The analyses that included RTMM-based adherence were only based on participants who were in the RTMM arm as RTMM was not used in the other arms.

To evaluate the ability of the various adherence measures to predict a detectable viral load, we conducted analyses using a cut-off of > 20 copies/mL. We calculated the sensitivity, specificity, positive predictive value (PPV) and negative predictive value (NPV) for different adherence cut-off values for each adherence assessment method separately. As previously studies suggested that current regimens are more forgiving for poor adherence than older regimens and that viral suppression can be achieved with 80% adherence, we classified participants as having poor adherence or good adherence using adherence cut-off values of 80%, 85%, 90%, 95% and 100% whereby the percentage stands for the percentage of doses taken. We used these to determine at which cut-off the prediction of a viral load ≥ 20 copies/mL was strongest.

For each of the adherence measures, its sensitivity was defined as the percentage of participants with a viral load ≥ 20 copies/ml who were identified by the methods as being poorly adherent at a certain adherence cut-off. Its specificity was defined as the percentage of participants with viral load < 20 copies/mL who were identified as being adherent at a certain adherence cut-off. The positive predictive value (PPV) was defined as the percentage of non-adherent participants with a detectable viral load, and the negative predictive value (NPV) as the percentage of adherent participants with an undetectable viral load.

The adherence measures were also combined to determine how two or three of them might impact sensitivity, specificity, PPV and NPV. To create composite adherence measures, participants were identified as non-adherent if they were below the adherence cut off in any of the combined measures under consideration. For example, when self-report and pharmacy refill counts were combined at a certain same cut-off and adherence was below 95% in either self-report or pharmacy refill count, the combined variable also was considered being below 95%.

For each of the adherence measures, sensitivity and (1-specificity) at the various adherence cut-off values were plotted in receiver operating characteristic (ROC) curves based on all the adherence data to determine the accuracy of an adherence measure to predict viral load. An Area under the ROC curve (AUC) value of 0.5 indicates that a test has no discriminatory capacity and an AUC of 1.0 indicates perfect discriminatory capacity. For screening purposes an AUC of 0.7 or higher is usually considered sufficient [30].

Besides the ROC curves analysis, logistic regression was used to identify which adherence measure predicted detectable a viral load ≥ 20 copies/mL while adjusting for gender and type of ART regimen. Analysis of baseline data of the parent trial had shown that TLE (the combination of tenofovir, lamivudine, efavirenz) was a significant predictor of viral load < 20copies/ml at study entry and therefore type of ARV regimen was categorized as TLE or another regimen. Two-sided p-values of < 0.05 were considered statistically significant in all analyses.

## Results

A total of 249 participants were enrolled and randomized and 233 (93.6%) completed the 48 weeks of the study with an available HIV viral load result at week 48. Of those, 161 participants (69%) had a viral load of < 20 copies/ml and 31% of the 233 had a viral load of ≥ 20 copies/ml at week 48. Of those with VL < 20copies/ml and with VL ≥ 20copies/ml, the majority (71.4–65%) were female, mean age were 43 years and 40 years, the median time since first known positive HIV test was 6.8 years and 8.5 years respectively. Furthermore, participants had used their current ART regimen for a median of 4.3 years and 4.7 years respectively. Most participants (76%) were using a first-line regimen which included efavirenz, nevirapine or dolutegravir and for eight participants the regimen was not recorded at week 48 (Table 1).

**Table 1:** Demographic and treatment characteristics of participants (N = 233)

Variable	N^*^	N (%) Mean(SD) Median (IQR)	Viral load < 20 copies/mL n = 161		Viral load ≥ 20 copies/mL n = 72	
			N^*^	Mean, Median SD/IQR/%	N^*^	Mean, Median SD/IQR/%
Sex						
Female	164	70.4%	117	71.3%	47	65%
Male	69	29.6%	44	63.8%	25	35%
Age		42(34–50)		43 (37–51)		40 (27.5–49)
Years since first positive HIV test Median (IQR)		7.2 (2.6–11.9)		6.8 (2.3–11.3)		8.5 (4.5–12.9)
Years on current ART Median (IQR)		4.4 (2.1–8.0)		4.3 (1.8–7.4)		4.7 (1.3–8.1)
ART Regimen						
NVP + AZT + 3TC	36	15%	23	14%	13	18%
EFV + TDF + 3TC	74	32%	60	37%	14	20%
EFV + TDF + FTC	13	6%	11	7%	2	3%
EFV + AZT + 3TC	18	8%	12	8%	6	8%
EFV + ATV						
EFV + ABC + 3TC						
ATV/r + AZT + 3TC	29	12%	11	7%	18	25%
ATV/r + TDF + FTC						
ATV/r + ABC + 3TC						
LPV/r + AZT + 3TC	18	8%	13	8%	5	7%
LPV/r + TDF + FTC						
LPV/r + ABC + 3TC						
DTG + 3TC + TDF	37	16%	26	16%	11	15%
DTG + AZT + 3TC						
DTG + ABC + 3TC						
Missing(ART)	8	3%	5	3%	3	4%

3TC, lamivudine; *ABC* abacavir, *ATV* atazanavir, *AZT* zidovudine, *EFV* efavirenz, *FTC* emtricitabine, *IQR* interquartile range, *LPV* lopinavir, *NPV* nevirapine, *r* ritonavir, *TDF* tenofovir disoproxil fumarate, *DTG* dolutegravir.

### Predictive value of individual adherence measures

In terms of the ability to predict a detectable viral load of ≥ 20 copies/ml, Table 1 shows that the *sensitivity* was lowest for self-reported adherence, higher for adherence by pharmacy refill counts and highest for adherence by RTMM at all adherence cut-off levels. Conversely, *specificity* was highest for self-reported adherence, lower for adherence by pharmacy refill counts and lowest for adherence by RTMM. The PPV ranged from 100 to 35% for self-reported adherence depending on the cut off value, while it was below 42% for adherence by pharmacy refill counts and RTMM. The NPV was consistently high (above 70%) for each of the measures at all cut-offs (see Appendix. Table 2).

**Fig. 1:**
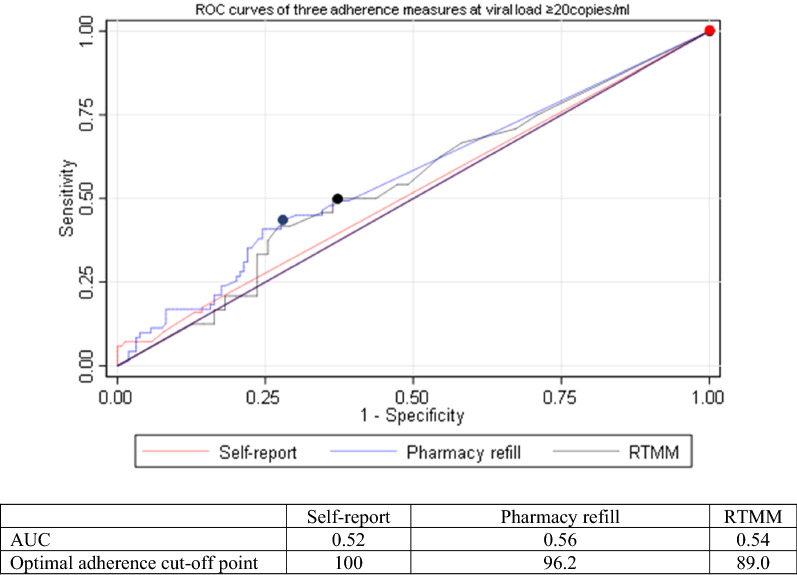
Receiver Operating Characteristic (ROC) curves for each assessment method (VL ≥ 20copies/ml) based on continuous adherence data

Overall, the AUCs of sensitivity versus 1-specificity of the individual adherence measures was lower than 0.7. Pharmacy refill and RTMM had AUC values of 0.56 and 0.54 respectively, while self-report had an AUC value of 0.52. The optimal adherence cut-off points, closest to the upper left part of the figure were 89% for RMM, 96.2% for pharmacy refill and 100% for self-report (see dots in Fig. 1).

Of the three measures, only self-reported adherence was significantly predicting viral load > 20 copies/ml at cut-offs of 85%, 90% and 95% in logistic regression analyses, after adjustment for gender and type of ART regimen. A regimen consisting of efavirenz, tenofovir and lamivudine, i.e., TLE, was a significant predictor of virological failure (p < 0.03). However, confidence intervals were wide, indicating low precision (see Appendix. Table 3).

### Predictive value of combinations of adherence measures

**Fig. 2:**
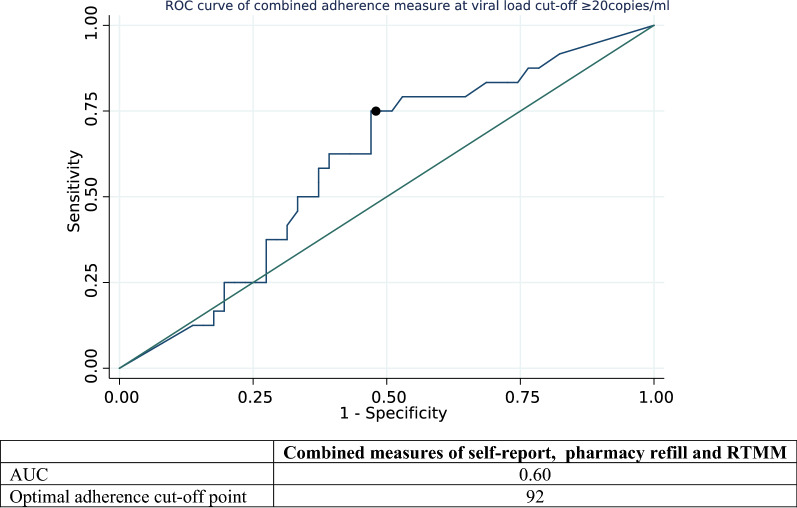
Receiver Operating Characteristic (ROC) curves for combined assessment method (VL ≥ 0copies/ml) based continuous adherence data

When we combined self-reported adherence and adherence by pharmacy refill count, there were no major difference in sensitivity and specificity compared to the individual measures (See Appendix Table 4). However, when all the three measures were combined, we observed a higher value of sensitivity and NPV at high cut-offs ranging from 95 to 100% (see Appendix Table 5: Combined measures at VL ≥ 20 copies/ml). The AUC for the combined measures slightly increased (0.60) as compared to the AUC values recorded among single-adherence measures (0.56, see Fig. 2). The optimal adherence cut-off point for the combined measures, closest to the upper left part of the figure, was 92% (see dots in Fig. 2).

In logistic regression models, the combined adherence measures did not significantly predict adherence at any adherence cut-off level **(**See Appendix Table 6). Furthermore, regardless of the cut-off used, being on a TLE regimen was the only independent predictor of a viral load < 20 copies/ml (see Appendix, Table 7).

## Discussion

This paper describes the accuracy by which three adherence measures, individually or in combination can predict virological failure, at different adherence cut-off levels. Overall, we found that adherence assessed exclusively by self-report, pharmacy refill count or RTMM were insufficiently sensitive to predict virological failure. Sensitivity markedly improved by combining all three measures, but the practical feasibility of such an approach would need to be studied.

We found that self-reported adherence had the lowest sensitivity, but the highest specificity to predict virological failure as compared to adherence assessed by pharmacy refill count and RTMM.

This implies that virological failure occurred in many participants despite a high level of self-reported adherence. This finding is in line with previous studies, which have shown that participants tend to overrate their adherence level. This likely reflects recall and/or social desirability bias and fear of being judged negatively by health care workers [22, 28]. Our finding that self-report had the highest specificity is in line with previous studies showing that reports of poor adherence can be trusted. Other advantages of self-reports are that they are relatively cheap and easy to implement in clinical practice [31–35].

All adherence measures investigated in the present study had areas under the ROC curves that were below 0.70, the minimal value for screening purposes, indicating insufficient ability to distinguish between patients with and without virological failure. Still, we observed that adherence by pharmacy refill counts and RTMM had showed a more promising performance compared with self-reported adherence. Similar findings of pharmacy refill adherence and RTMM having higher AUC values than self-report methods were observed in studies conducted in Tanzania and Botswana [32, 36, 37]. Other studies also found that an electronic monitoring device had a higher sensitivity in predicting virological failure compared to self-report for participants with > 80% adherence [10, 38].

Therefore, in the context of routine clinical practice, where RTMM is not yet available, or is quite expensive, our findings demonstrate that pharmacy refill counts could provide a better prediction of virological failure given its higher sensitivity compared to self-reported adherence. In addition, RTMM could be cost-effective in a context of differentiated service delivery, i.e. if prioritized for use in poorly adherent participants. This could be particularly relevant in settings were viral load monitoring is not available [39].

When we combined the three measures, we observed the measures performed better compared to individual measures as the highest sensitivity and PPV were recorded at a higher range of cut-offs (95–100%) for viral load of > 20 copies/mL. Our finding that the optimal adherence cut-off to predict VL failure was around 90% or more is consistent with the WHO’s guidance that achieving a degree of adherence to ART of 95% reduces both the emergence of antiretroviral drug resistance and the risk of HIV disease progression.[40]. The ROC curve of combined adherence measures indicated a slightly higher AUC value compared to single-adherence measures.

Our results imply that where possible, existing adherence methods need to be combined to obtain a more comprehensive assessment of adherence as each adherence assessment method may capture a different aspect of medication taking behaviour and will give a better prediction of virological failure [38, 41]. Our findings also imply that self-reports of poor adherence should be taken seriously, and that patients reporting poor adherence might benefit from adherence counselling and intervention.

This study has some limitations. First, each adherence measure has its own specific limitations that may have affected the results. For RTMM, we assumed that all the openings of the device indicated intake of the medication by participants. We are aware that medication may not have been taken, but rather shared, or dumped [42]. Moreover, the Wisepill^®^ device occasionally lost connectivity with the server and failed to record intake data on time, as participants had forgotten to charge the device. Second, the sample size was small, particularly concerning the number of participants using the RTMM device (one-third of the total trial population) which has likely limited our ability to predict virological failure. Hence, our results should be considered exploratory. Third, the study was conducted in only two clinics from the urban Kilimanjaro region, limiting the generalizability of the study outcomes, e.g. to rural populations of PLHIV. Fourth, the results of self-report and pill counts may have been influenced by recall bias, other errors (e.g., miscalculation) and/or self-interpretation by the participants. The use of laboratory methods to detect plasma drug levels might have resulted in an improved AUC to predict detectable viral load [43]. Finally, whereas WHO endorses the use of a linear visual analogue scale (VAS) to potentially reduce bias in assessing self-reported adherence [44], we chose to use the self-reported adherence measure in the manner it is currently most commonly used as part of standard of care in Tanzania.

The strength of this paper is that, to our knowledge this is the first study that compared the performance of three adherence measures in the context of a randomized clinical trial. Also, our findings included both manually recorded data (self-report, pharmacy refill) during clinic visits and electronically (automated real time data from the Wisepill box). This allowed us to compare and identify potential discrepancies between the data sources as described previously [45].

## Conclusion

Our results show that adherence assessed by either self-report, pharmacy refill count or RTMM on its own did not perform well in predicting virologic failure. This could potentially be improved by combining all three measures, but the practical feasibility of such an approach would need to be studied. Given the fact that we had a small sample size, particularly considering the number of participants using the RTMM device, we would encourage researchers to investigate the same in bigger studies in order to be able to have adequate power for conclusions. In a context where RTMM is not available, our data show that pharmacy refill adherence could provide a better prediction of virological failure than self-report, but that reports of poor adherence should be taken seriously.

## Data Availability

All the Data are available and will be shared after signing of the Data Transfer agreement between Sender and Receiver.

## Appendix

See Appendix Tables 2, 3, 4,5, 6, 7

**Table 2:** Sensitivity, specificity, PPV and NPV of self-report, pharmacy refill and RTMM at viral load ≥ 20 copies/ml

Cut-off %						
Adherence measure	Viral load		Sensitivity	Specificity	PPV	NPV
	≥ 20	< 20				
Self-report						
< 80%	4	0	5.8	100	100	70.3
≥ 80%	65	154				
Pharmacy refill						
< 80%	14	26	19.7	83.6	35.0	70.0
≥ 80%	57	133				
RTMM						
< 80%	10	16	41.7	70.9	38.5	73.6
≥ 80%	14	39				

Cut-off 85%						
	Viral load		Sensitivity	Specificity	PPV	NPV
	≥ 20	< 20				
Self-report						
< 85%	4	1	5.8	99.3	80.0	70.2
≥ 85%	65	153				
Pharmacy refill						
< 85%	17	29	23.9	81.8	37.0	70.6
≥ 85%	54	130				
RTMM						
< 85%	11	19	45.8	65.4	36.7	73.5
≥ 85%	13	36				

Cut-off 90%						
	Viral load		Sensitivity	Specificity	PPV	NPV
	≥ 20	< 20				
Self-report						
< 90%	4	1	5.8	99.3	80.0	70.2
≥ 90%	65	153				
Pharmacy refill						
< 90%	21	34	29.6	78.6	38.2	71.4
≥ 90%	50	125				
RTMM						
< 90%	12	22	50.0	60.0	35.3	73.3
≥ 90%	12	33				

Cut-off 95%						
	Viral load		Sensitivity	Specificity	PPV	NPV
	≥ 20	< 20				
Self-report						
< 95%	5	8	7.2	94.8	38.5	69.5
≥ 95%	64	146				
Pharmacy refill						
< 95%	29	40	40.8	74.8	42.0	73.9
≥ 95%	42	119				
RTMM						
< 95%	13	26	54.2	52.7	33.3	72.5
≥ 95%	11	29				

Cut-off 100%						
	Viral load		Sensitivity	Specificity	PPV	NPV
	≥ 20	< 20				
Self-report						
< 100%	12	22	17.4	85.7	35.3	69.8
100%	57	132				
Pharmacy refill						
< 100%	35	62	49.3	61.0	36.1	72.9
100%	36	97				
RTMM						
< 100%	18	39	75.0	29.1	31.6	72.7
100%	6	16				

*PPV* positive predict value, *NPV* negative predict value, *RTMM* real time medication monitoring.

**Table 3:** Logistic regression model results of different adherence measures predicting a detectable viral load of ≥ 20 copies/ml, at different adherence cut-offs

Viral load ≥ 20 copies/ml			AOR(95%CI)	P
Self-report	** ≥ 80%**			–
	** < 80%**	**Ref**		
Pharmacy refill	** ≥ 80%**		0.8 (0.4–1.8)	0.679
	** < 80%**		1	
RTMM	** ≥ 80%**		0.6 (0.2–1.7)	0.322
	** < 80%**	**Ref**	1	
Self-report	** ≥ 85%**		0.1 (0.01–1.0)	0.053
	** < 85%**	**Ref**	1	
Pharmacy refill	** ≥ 85%**		0.8 (0.4–1.5)	0.454
	** < 85%**	**Ref**	1	
RTMM	** ≥ 85%**		0.7 (0.2–2.0)	0.508
	** < 85%**	**Ref**	1	
Self-report	** ≥ 90%**		0.1 (0.01–1.0)	0.053
	** < 90%**	**Ref**	1	
Pharmacy refill	** ≥ 90%**		0.7 (0.4–1.4)	0.365
	** < 90%**	**Ref**	1	
RTMM	** ≥ 90%**		0.7 (0.2–1.9)	0.465
	** < 90%**	**Ref**	1	
Self-report	** ≥ 95%**		0.6 (0.2–2.1)	0.477
	** < 95%**	**Ref**	1	
Pharmacy refill	** ≥ 95%**		0.5 (0.3–1.0)	0.051
	** < 95%**	**Ref**	1	
RTMM	** ≥ 95%**		0.7 (0.2–1.9)	0.492
	** < 95%**	**Ref**	1	
Self-report	**100%**		0.7 (0.3–1.5)	0.356
	** < 100%**	**Ref**	1	
Pharmacy refill	**100%**		0.7 (0.4–1.2)	0.190
	** < 100%**	**Ref**	1	
RTMM	**100%**		0.8 (0.2–2.6)	0.727
	** < 100%**	**Ref**	1	

*Ref* reference category

*AOR* Odds ratios adjusted for gender and ART regimen.

*RTMM* real time medication monitoring.

**Table 4:** Sensitivity, Specificity, NPV and PPV for combined self-reported and pharmacy refill adherence

Cut-offs	Viral load		Sensitivity	Specificity	PPV	NPV
	≥ 20	< 20				
< 80%	14	26	20.3	83.2	35.0	70.1
≥ 80%	55	129				
< 85%	17	29	24.6	81.3	37.0	70.8
≥ 85%	52	126				
< 90%	21	34	30.0	78.1	38.2	71.2
≥ 90%	49	121				
< 95%	30	45	42.9	71.0	40.0	73.3
≥ 95%	40	110				
< 100%	40	74	57.1	52.6	35.1	73.2
100%	30	82				

*PPV* positive predict value, *NPV* negative predict value, *RTMM* real time medication monitoring.

**Table 5:** Sensitivity, Specificity, NPV and PPV for combined self-report, pharmacy refill and RTMM adherence measure

Cut-offs	Viral load		Sensitivity	Specificity	PPV	NPV
	≥ 20	< 20				
< 80%	11	20	45.8	63.0	35.5	72.3
≥ 80%	13	34				
< 85%	14	22	58.3	59.3	38.9	76.2
≥ 85%	10	32				
< 90%	15	26	62.5	52.7	36.6	76.3
≥ 90%	9	29				
< 95%	19	33	79.2	40.0	36.5	81.5
≥ 95%	5	22				
< 100%	22	46	91.7	16.4	32.3	81.8
100%	2	9				

*PPV* positive predict value, *NPV* negative predict value, *RTMM* real time medication monitoring.

NB: N is small due to RTMM being part of this analyses and a small percentage of participants used RTMM.

**Table 6:** Logistic regression model results of all three adherence measures combined predicting viral load of ≥ 20 copies/ml, at different adherence cut-offs

Adherence	AOR (95%CI)	P
** ≥ 80%**	0.78(0.28–2.18)	0.64
< 80%	**1**	
** ≥ 85%**	0.60(0.215–1.72)	0.345
< 85%	1	
** ≥ 90%**	0.65 (0.23–1.82)	0.410
< 90%	1	
** ≥ 95%**	0.43 (0.134–1.37)	0.158
< 95%	1	
**100%**	0.55 (0.098–3.07)	0.496
< 100%	1	

*AOR* adjusted odds ratio, adjusted for gender and ART regimen

**Table 7:** Logistic regression models showing ART regimen and gender at different adherence cut-offs predicting detectable viral load at cut-off of ≥ 20 copies/ml

Viral load ≥ 20 copies/ml			AOR (95%CI)	P
Self-report ≥ **80%**	TLE		0.4(0.2–0.9)	0.019
	Other regimens	**Ref**	1	
	Female		0.8(0.4–1.5)	**0.5**
	Male	**Ref**	1	
Pharmacy refill ≥ **80%**	TLE		0.4 (0.2–0.8)	0.012
	Other regimens	**Ref**	1	
	Female		0.8(0.5–1.6)	0.6
	Male	**Ref**	1	
RTMM ≥ **80%**	TLE		0.2(0.06–0.8)	0.025
	Other regimens	**Ref**	1	
	Female		0.7(0.25–2.2)	0.59
	Male	**Ref**	1	
Self-report** ≥ 85%**	TLE		0.4(0.2–0.8)	0.011
	Other regimens	**Ref**	1	
	Female		0.11(0.12–1.02)	0.65
	Male	**Ref**	1	
Pharmacy refill ≥ **85%**	TLE		0.4(0.2–0.8)	0.014
	Other regimens	**Ref**	1	
	Female		0.77(0.4–1.5)	0.622
	Male	**Ref**	1	
RTMM ≥ **85%**	TLE		0.22(0.06–0.84)	0.03
	Other regimens	**Ref**	1	
	Female		0.7(0.25–1.97)	0.54
	Male	**Ref**	1	
Self-report ≥ **90%**	TLE		0.4(0.2–0.8)	0.011
	Other regimens	**Ref**	1	
	Female		0.9(0.46–1.6)	0.65
	Male	**Ref**	1	
Pharmacy refill ≥ **90%**	TLE		0.4(0.2–0.8)	0.014
	Other regimens	**Ref**	1	
	Female		0.85(0.46–1.6)	**0.63**
	Male	**Ref**	1	
RTMM ≥ **90%**	TLE		0.1(0.06–0.84)	0.028
	Other regimens	**Ref**	1	
	Female		0.7(0.2–2.04)	0.52
	Male	**Ref**	1	
Self-report ≥ **95%**	TLE		0.2(0.2–0.8)	0.008
	Other regimens	**Ref**	1	
	Female		0.9 (0.5–1.65)	0.7
	Male	**Ref**	1	
Pharmacy refill ≥ **95%**	TLE		0.3(0.2–0.9)	0.017
	Other regimens	**Ref**	1	
	Female		0.9 (0.5–1.7)	0.72
	Male	**Ref**	1	
RTMM ≥ **95%**	TLE		0.2 (0.06–0.8)	0.024
	Other regimens	**Ref**	1	
	Female		0.68 (0.24–1.9)	0.5
	Male	**Ref**	1	
Self-report **100%**	TLE		0.3 (0.2–0.8)	0.007
	Other regimens	**Ref**	1	
	Female		0.87 (0.5–1.6)	0.7
	Male	**Ref**	1	
Pharmacy refill **100%**	TLE		0.4(0.2–0.8)	0.01
	Other regimens	**Ref**	1	
	Female		0.8(0.47–1.64)	0.7
	Male	**Ref**	1	
RTMM **100%**	TLE		0.2(0.05–0.8)	0.025
	Other regimens	**Ref**	1	
	Female		0.68(0.2–1.97)	0.48
	Male	**Ref**	1	

*TLE* Tenofovir/Lamivudine/Efavirenz.

Other regimen: *ABC* abacavir, *ATV* atazanavir, *AZT* zidovudine, *FTC* emtricitabine, *IQR* interquartile range, *LPV* lopinavir, *NPV* nevirapine, *r* ritonavir, *DTG* dolutegravir, *Ref* reference category.
